# Telemedical education during national emergencies: learning from Kashmir

**DOI:** 10.1111/tct.13204

**Published:** 2020-06-30

**Authors:** Bushra S Imtiyaz, Elisabeth A Garratt, Joanna H Cox, Roxanne C Keynejad

**Affiliations:** ^1^ Department of Psychiatry Government Medical College Srinagar Jammu and Kashmir India; ^2^ Sheffield Methods Institute University of Sheffield Sheffield UK; ^3^ Sandwell General Hospital, Lyndon West Bromwich West Midlands UK; ^4^ Section of Women's Mental Health Department of Health Service and Population Research Institute of Psychiatry Psychology& Neuroscience King's College London London UK

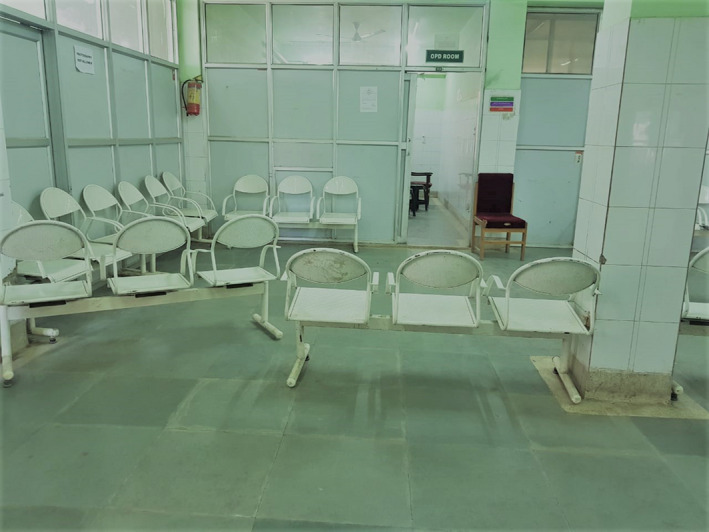

The coronavirus pandemic of 2020 has highlighted both similarities and vast inequalities between nations. Periods of preparation, lockdown and recovery from COVID‐19, staggered across regions, have refocused national priorities on strengthening health system capacity, scaling up acute care, future‐proofing economies and adapting education for remote delivery.

Disruption to formal education is not unusual in countries experiencing multiple continuing crises. Regions affected by both geoclimatic vulnerability and chronic political unrest regularly experience interruptions to health care and education provision. Between 2016 and 2017, the schools and colleges of Kashmir, northern India, were closed for 60% of all working days. The region is currently nearing a full year under lockdown.

High‐quality medical education remains essential to health care delivery, especially during pandemics and periods of geopolitical and sociopolitical unrest

High‐quality medical education remains essential to health care delivery, especially during pandemics and periods of geopolitical and sociopolitical unrest. COVID‐19 has increased the global recognition of health care workers, highlighting the need for uninterrupted clinical education. With nations forced to recruit medical and nursing students to deliver frontline care, rapidly expand bed capacity and share ventilators, the coronavirus pandemic has exposed the scarcity of health care resources globally. Low‐ and middle‐income countries (LMICs) are especially vulnerable and most in need of continued remote education and training.

In the long term, frequent disruption to health care education can impact clinical standards and encourage a ‘brain drain’ of skilled professionals to high‐income regions. Innovative pedagogical approaches are required to fill gaps in medical education, especially during global health emergencies such as COVID‐19. E‐learning has been recognised by the World Health Organization (WHO) as an important means of addressing health workers’ educational needs, especially in LMICs.^1^ Remotely delivered psychiatry education is particularly necessary for the many regions where the provision of mental health care is sparse. A recent *Lancet* commission emphasised the benefits to global mental health of digital technologies, for the training and supervision of health workers, as well as for the provision of care in the most remote and underserved regions.^2^ Online methods pose as many challenges as solutions, however. Massive open online courses (MOOCs) have particularly low completion rates, attributed to the lack of personal contact, limited feedback or monitoring that learners receive.^3^ If the future of medical education must be at least partly online, how can e‐learning initiatives maximise their appeal to learners and ensure continued engagement?

Innovative pedagogical approaches are required to fill gaps in medical education, especially during global health emergencies such as COVID‐19

Peer learning can enhance medical education when senior faculty members must prioritise clinical care. A peer‐to‐peer e‐learning partnership (‘Aqoon’) between medical students from Somaliland and the UK demonstrated reciprocal knowledge and cross‐cultural benefits.^4^ The model, employing the low‐bandwidth MedicineAfrica website, has been iteratively refined for the post‐conflict Somaliland context since 2010, and continues to evolve. Could Aqoon be applicable to learners in a dramatically different setting, facing unique barriers to continued education (Boxes 1 and 2)?

Box 1 **Peer‐to‐peer e‐learning between Kashmir and UK medical students**

- We piloted a global mental health e‐learning partnership between volunteer medical students in Kashmir and the UK in 2015. Kashmir was gradually rebuilding after severe flooding in 2014 and a major earthquake in 2005. We guided five pairs of student volunteers to meet online for 10 fortnightly, hour‐long, peer‐led tutorials covering topics from the WHO mental health gap action programme (mhGAP) intervention guide. We used fortnightly e‐mails to remind partners to read specified mhGAP chapters covering core psychiatric presentations before discussing their understanding and clinical experience online, in pairs.
- We used pre‐ and post‐course questionnaires to evaluate the programme and elicit student reflections (Box 2). Kashmir students gained confidence and knowledge about clinical presentations, diagnosis and treatment options. One said that they ‘now better appreciate the role the society can play with regards to management and rehabilitation’. UK learners gained opportunities for examination revision and understanding of cross‐cultural differences in clinical presentations, help‐seeking, management, education and health systems. Ninety percent of students would recommend the programme to a friend and one Kashmiri participant went on to pursue specialty psychiatry training. The model benefitted from the MedicineAfrica platform's design for low bandwidths; however, the requirement for reliable internet was a key limitation.

Box 2 **Learner reflections on Aqoon**

**Kashmir**

Online discussions with my UK peer helped me to gain a deeper understanding of cross‐cultural mental health scenarios. I reflected on the ways that conflict and cultural beliefs can impact mental health. This experience made me appreciate the protective value of Kashmir's family systems, and cultural and religious beliefs, as well as the scope to improve the delivery of mental health care services. My experience with this project influenced me to pursue psychiatry training in Kashmir.

**UK**

Discussing different [WHO mental health gap action programme] mhGAP topics with my partner in Kashmir helped to consolidate my knowledge of symptoms, diagnosis and management strategies for common psychiatric conditions. Perhaps more importantly, we discussed the position of mental health within our respective health care systems. I found this aspect of the partnership particularly instructive. The experience has helped me to appreciate our mental health service infrastructure, despite its limitations.

Frequent internet and communication blackouts in conflict regions may be the biggest impediment to their feasibility. Internet access in Kashmir remains restricted to low‐speed 2G services, impacting the general population and health care professionals’ ability to keep up to date with clinical guidelines and research developments. Where connectivity, electricity and the availability of computers and smartphones are limited, more conventional alternatives may be required. Radio and television broadcasts lack the real‐time interaction, bi‐directional communication and instant feedback of a simulated classroom environment, however. User‐friendly interfaces, versatility and familiarity to learners make online approaches preferable in settings where the internet is reliable. Political commitment to ensure fast and continuous internet connectivity, at least for professionals working in essential services and delivering clinical education, is increasingly important.

Digital literacy and the availability of affordable technologies impact the accessibility of online education. Three years after the flagship ‘digital India’ launch, India was one of the world's most rapidly digitising nations. In China, the Ministry of Education has directed major telecommunication service providers to boost internet connectivity and upgrade the bandwidth of online education service platforms in the wake of COVID‐19. Similarly, telecommunication companies in Croatia are providing free internet access to students of low socio‐economic status.

Teaching methods must be flexible if they are to ensure the continuity of medical education during turbulent times. E‐learning models represent low‐cost innovations that can be customised to the clinical and pragmatic context. Peer learning has the potential to benefit learners in more‐ and less‐resourced settings alike, whilst the resurgence in volunteerism provoked by COVID‐19 could see skilled, knowledgeable professionals who are unable to deliver front‐line care instead giving their time and expertise for remote education. Such initiatives require the same rigor and quality standards as any face‐to‐face teaching.

COVID‐19 could see skilled, knowledgeable professionals who are unable to deliver front‐line care instead giving their time and expertise for remote education

The coronavirus pandemic presents challenges and opportunities. One is to develop the evidence base for e‐learning models using randomised methods in larger samples, to determine their scalability and suitability to current needs. Lessons learned in the aftermath of emergencies can benefit long‐term practice and preparedness to respond to future upheaval.^5^ Although the first priority of COVID‐19 must be clinical care, the need to strengthen health systems, health professionals and clinical education must not be neglected.

## References

[1] World Health Organization . eLearning for undergraduate health professional education – a systematic review informing a radical transformation of health workforce development. 2015. Available at https://www.who.int/hrh/docum​ents/14126-eLear​ningR​eport.pdf?ua=1. Accessed on 05 June 2020.

[2] Patel V , Saxena S , Lund C , et al. The Lancet Commission on global mental health and sustainable development. Lancet 2018;392(10157):1553–1598. 30314863 10.1016/S0140-6736(18)31612-X

[3] Atiaja L , Proenza R . The MOOCs: origin, characterization, principal problems and challenges in higher education. Journal of e‐Learning and Knowledge Society 2016;12(1):65–76.

[4] Keynejad R . Global health partnership for student peer‐to‐peer psychiatry e‐learning: lessons learned. Globalization and Health 2016;12:82. 27912763 10.1186/s12992-016-0221-5 PMC5135829

[5] World Health Organization . Building back better: Sustainable mental health care after emergencies. Available at https://apps.who.int/iris/bitst​ream/handl​e/10665/​85377/​97892​41564​571_eng.pdf. Accessed on 05 June 2020.

